# Comparative effectiveness of virtual reality (VR) vs 3D printed models of congenital heart disease in resident and nurse practitioner educational experience

**DOI:** 10.1186/s41205-022-00164-6

**Published:** 2023-02-11

**Authors:** Jonathan Awori, Seth D. Friedman, Christopher Howard, Richard Kronmal, Sujatha Buddhe

**Affiliations:** Division of Pediatric Cardiology and Radiology, Seattle Children’s Hospital, Seattle, WA USA

**Keywords:** 3D printed models, Virtual reality, Congenital heart disease, Education

## Abstract

**Background:**

Medical trainees frequently note that cardiac anatomy is difficult to conceive within a two dimensional framework. The specific anatomic defects and the subsequent pathophysiology in flow dynamics may become more apparent when framed in three dimensional models. Given the evidence of improved comprehension using such modeling, this study aimed to contribute further to that understanding by comparing Virtual Reality (VR) and 3D printed models (3DP) in medical education.

**Objectives:**

We sought to systematically compare the perceived subjective effectiveness of Virtual Reality (VR) and 3D printed models (3DP) in the educational experience of residents and nurse practitioners.

**Methods:**

Trainees and practitioners underwent individual 15-minute teaching sessions in which features of a developmentally typical heart as well as a congenitally diseased heart were demonstrated using both Virtual Reality (VR) and 3D printed models (3DP). Participants then briefly explored each modality before filling out a short survey in which they identified which model (3DP or VR) they felt was more effective in enhancing their understanding of cardiac anatomy and associated pathophysiology. The survey included a binary summative assessment and a series of Likert scale questions addressing usefulness of each model type and degree of comfort with each modality.

**Results:**

Twenty-seven pediatric residents and 3 nurse practitioners explored models of a developmentally typical heart and tetralogy of Fallot pathology. Most participants had minimal prior exposure to VR (1.1 ± 0.4) or 3D printed models (2.1 ± 1.5). Participants endorsed a greater degree of understanding with VR models (8.5 ± 1) compared with 3D Printed models (6.3 ± 1.8) or traditional models of instruction (5.5 ± 1.5) *p* < 0.001. Most participants felt comfortable with modern technology (7.6 ± 2.1). 87% of participants preferred VR over 3DP.

**Conclusions:**

Our study shows that, overall, VR was preferred over 3DP models by pediatric residents and nurse practitioners for understanding cardiac anatomy and pathophysiology.

**Supplementary Information:**

The online version contains supplementary material available at 10.1186/s41205-022-00164-6.

## Introduction

Medical trainees frequently note that cardiac anatomy and pathophysiology is difficult to fully conceive in a two dimensional (2D) framework [1, 2]. While it is certainly possible to extrapolate three dimensional (3D) relationships from 2D representations, this conceptual leap is best achieved with an acuity developed over time, not readily available to the novice learner. Such an understanding is especially important in pediatric cardiology in which there is an intricate relationship between the spatial orientation of cardiac anatomy and associated physiology. In an attempt to strengthen this understanding, various 3D modalities have been developed including 3D digital models (3DD), 3D printed models (3DP), Virtual Reality (VR), Augmented Reality (AR), and Mixed reality (MR) [3–5]. VR is an immersive digital experience in a simulated environment separate from the real world typically using a head-mounted display or headset. AR enhances or augments a real world environment with superimposed digital information such as data and images while MR extends AR to allow for interaction between the virtual and real word components of the combined environment [6]. While the use of each of these modalities is growing substantially, there is sparse data on the comparative value of each modality.

3DP has found multiple applications in pediatric cardiology education. Several studies have incorporated 3DP into curricula to compare learner response compared to traditional teaching modalities [1, 7–9]. These studies have consistently found improved learner satisfaction in the domains of knowledge acquisition and structural conceptualization. Of increasing interest has been whether such subjective assessments translate into improved objective performance. One study compared pre-test and post-test performance for a control group (traditional teaching) versus intervention group (3DP) in relation to knowledge acquisition about vascular rings [9]. This study found improved performance for the intervention group. Another study, similarly structured, found no difference in post-scores for medical student groups exposed to traditional 2D vs 3D printed models of tetralogy of Fallot [1]. The authors speculated, however, that the lack of improvement may have been due to questions that focused on pre-formed medical knowledge versus the spatial orientation assessment that 3D printed models would be more likely to enhance.

While 3DP has found several landing points in medical education, surgical preparation, and clinical reinforcement, 3DP has important limitations including cost and limited cutting planes. Significant interest has therefore developed in alternative 3D dimensional representations including Virtual Reality (VR), Augmented Reality (AR) and Mixed Reality (MR) [5, 10]. Sacks and Axelrod (2020) connect adult learning theory to the potential pedagogical value of VR by noting that adults learn best when they are in control of their learning environment, a framework that is congruent with the interactive space of VR [11]. In a study that compared understanding of congenital heart disease among residents and medical students exposed to VR versus conventional 2D display, improved diagnostic scores were noted among the intervention group [2]. Building on the capacity of VR to shorten preparation times, another study demonstrated that applying VR directly to raw MRI data without intermediate segmentation steps could shorten preparation time to 5 min compared to the 8 hours for 3DP [12].

A comparative study in the field of neurosurgery found VR was more effective than 3DP and traditional 2D representations in enhancing understanding of craniovertebral junction deformities [13]. Similarly, in a comparative study including multi-level trainees, VR angiograms have been found to outperform 3DP in regard to resolution, ability to zoom and ease of manipulation while 3DP had the advantage in depth perception [14]. In the context of congenital heart disease, a comparative study between VR and 3DP found similar subjective assessments of visual clarity between 3DP and VR but greater perceived instructive potential for VR over 3DP among the participants composed exclusively of radiographers, sonographers and radiologists [15] To our knowledge, there has not been a study directly comparing the utility of VR versus 3DP in CHD education among medical trainees. Our study sought to make this comparison by giving trainees an opportunity to interact with both representations back to back followed by an assessment of their relative effectiveness in enhancing their understanding of normal heart architecture as well as a common CHD lesion, tetralogy of Fallot.

## Materials and methods

**Fig. 1:**
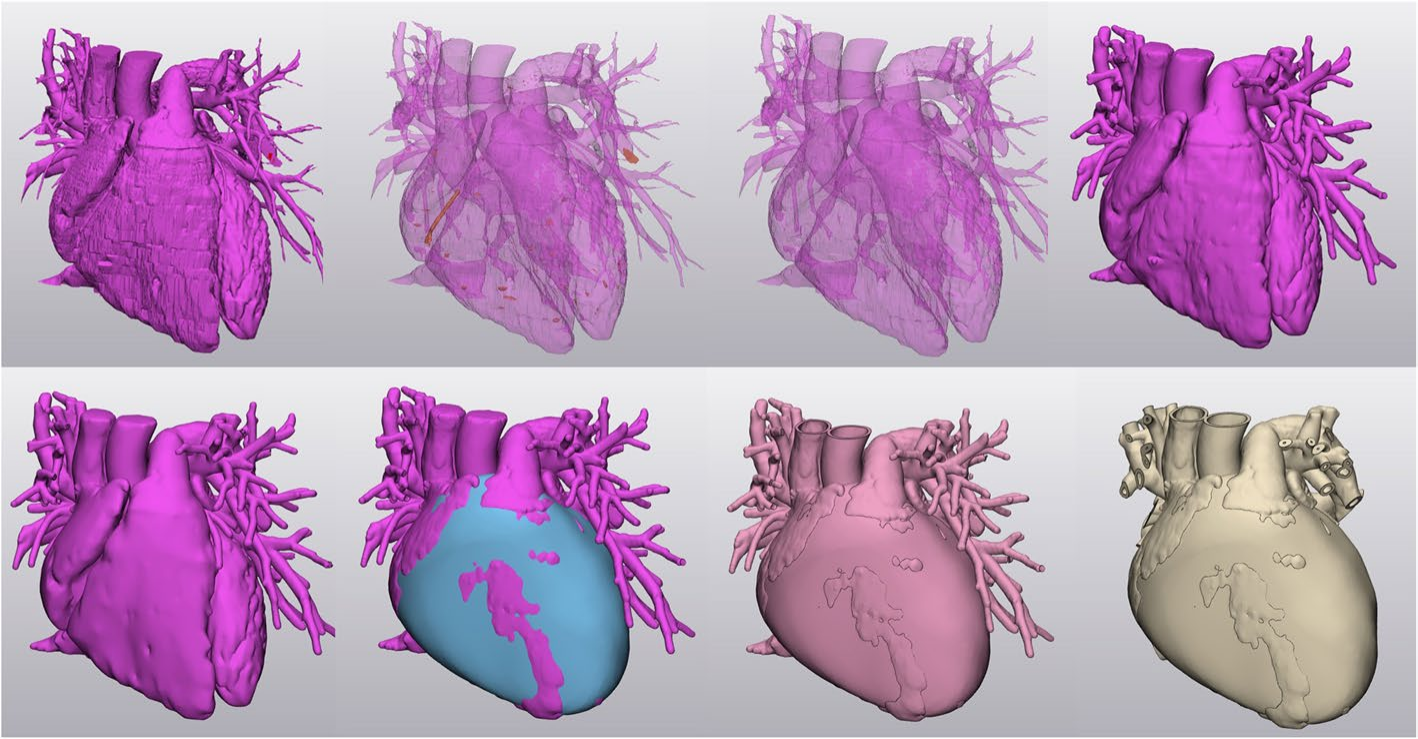
Model Preparation. At top left, the raw blood pool is seen. Islands are selected (orange regions), removed, and the shell is wrapped (top right). A hollowed outer surface is created (bottom left), to which a myocardium is added (blue). These parts undergo Boolean union, and then are progressively trimmed to yield the final whole heart model (bottom right)

As an initial step to create the 3D models, cardiac CT/MRI data for a developmentally typical heart (15 year old patient) and tetralogy of Fallot (15 year old patient) were identified from our institutional cross sectional imaging database. Raw DICOM data from either MRI or CT was loaded into MIMICS (version 19, Materialise, Leuven Belgium) and segmented to label the blood pool and myocardium. Objects were generated and exported to 3-MATIC (version 11, Materialise, Leuven Belgium) for the following steps: wrapping, island removal, smoothing, exterior hollowing, Boolean union (blood pool’s derived shell with the myocardium), vessel trimming to provide a visually and ambiguous heart and slicing into parts to ensure that the goal features of anatomy would be easily visualized (Fig. 1). More than one color was used but these were divided along opening planes, rather than by anatomical components to limit potential visual distraction away from the defect.

IRB approval was obtained in order to obtain patient data. Patients with the above congenital heart disease were identified from our CT/MR database generating the. STL file. The appropriate model was then created. For these hearts, cut planes were determined to ensure that the anatomical features were abundantly clear and unambiguous to the viewer with minimal visual exploration. The 3D model data was then overlaid on the native data to assess for accuracy. Following cut-plane selection, models were scaled to be of similar size and columnar punchouts were created on the cut faces. This facilitated post-printing embedding of magnets to allow the models to “snap” together.

**Fig. 2:**
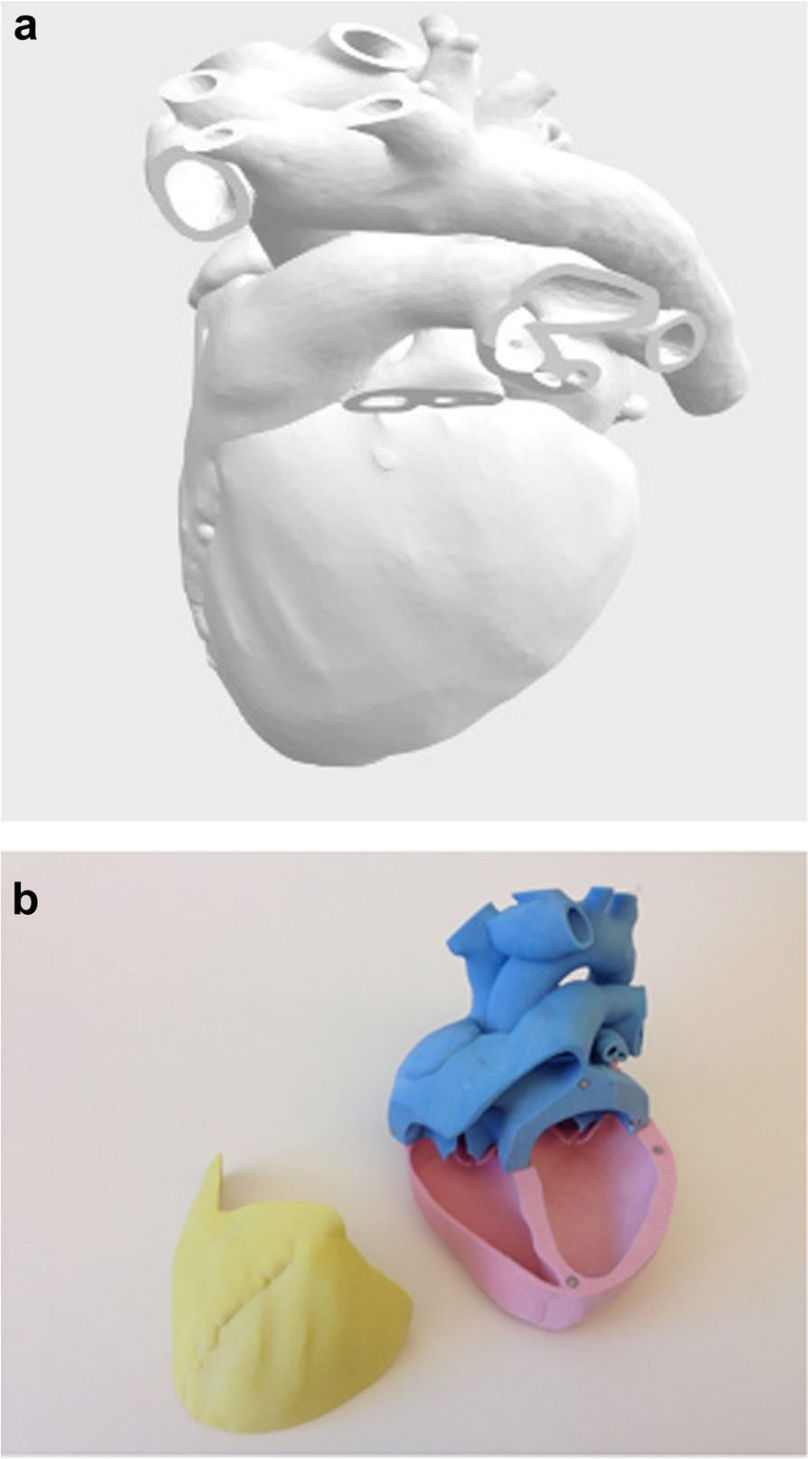
**a** Developmentally typical heart Grey scale VR model. **b** Developmentally typical heart Color 3D printed model

**Fig. 3:**
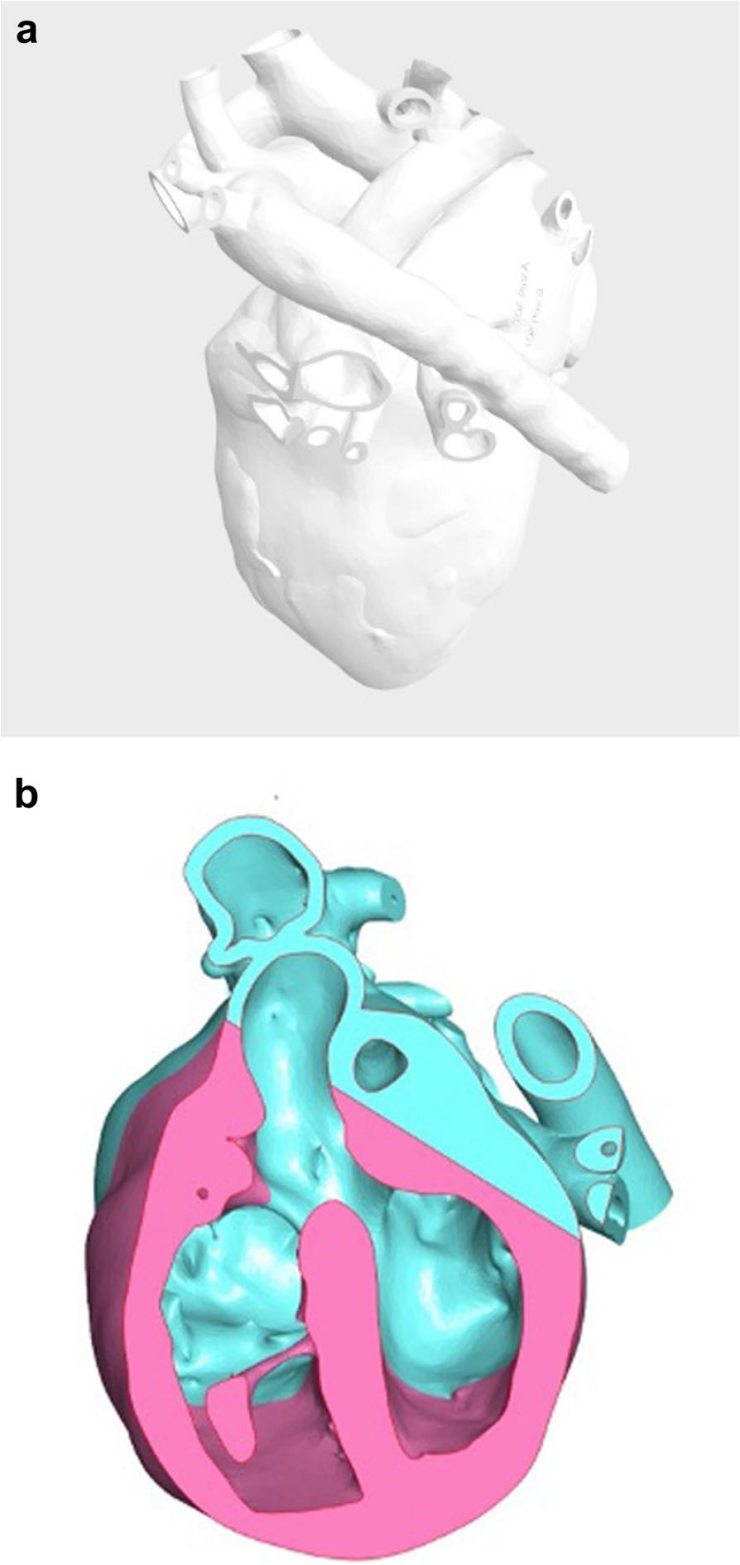
**a** Tetralogy of Fallot Grey scale VR model. **b** Tetralogy of Fallot Color VR model

STL models were first printed on a ZPrinter®250 printer (Z-Corp, Cambridge, MA) with cyanoacrylate infiltration. As thin sections such as valve component or vessels branches remained fragile, models were reprinted in multi-jet fusion (MJF 580, Hewlett-Packard, Palo Alto CA). Then, magnets were placed, the parts were selectively dyed with conventional fabric dye, and employed for the described work. Segmentation and post-processing were performed by a trained and experienced pediatric cardiologist (SB) and imaging scientist (SDF) Figs. 2 and 3.

**Fig. 4:**
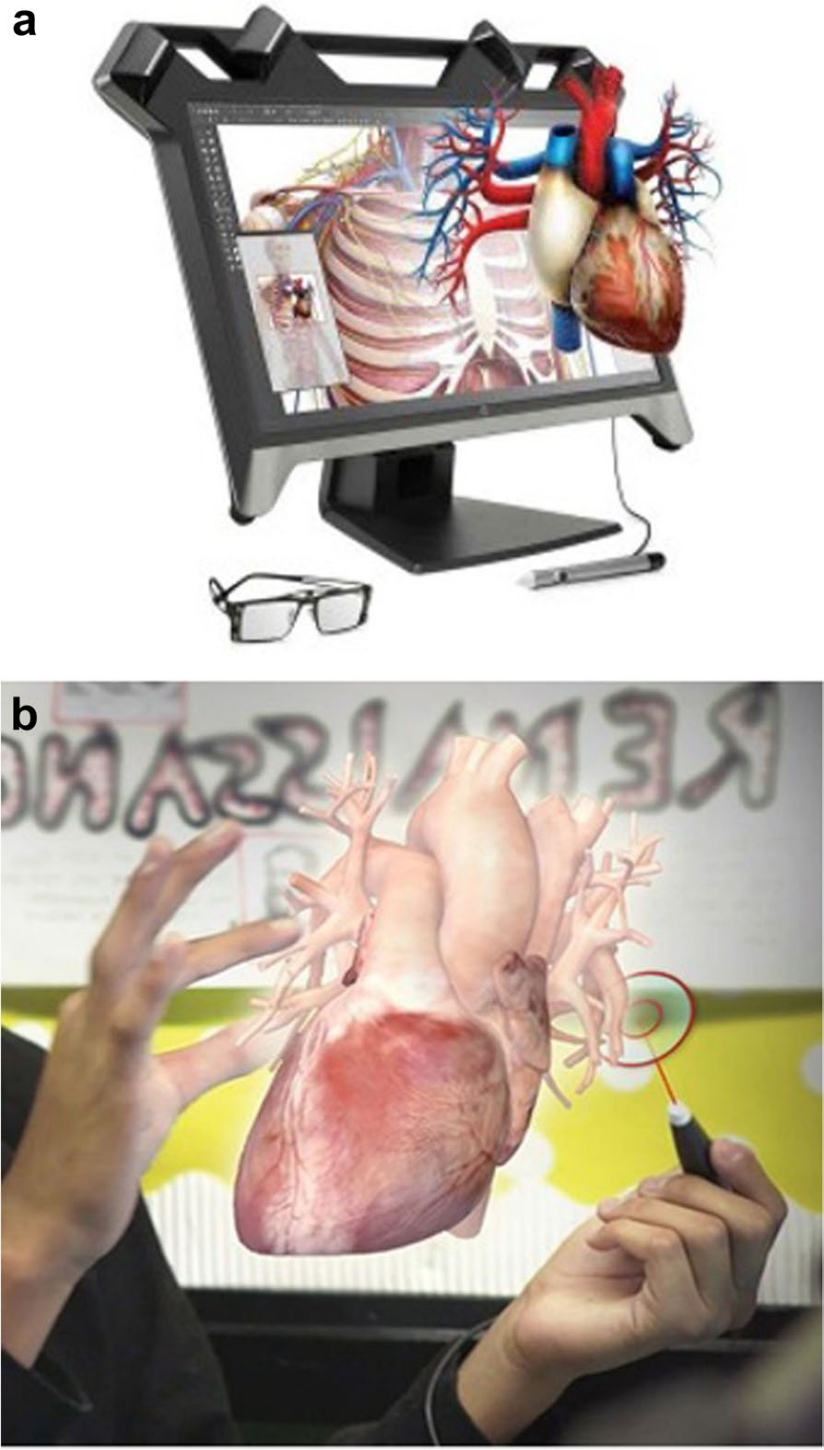
**a** VR Console (photo credit zSpace®). **b** VR Wand (photo credit zSpace®)

The VR models were derived from the same models created from the CT/MRI data used to form the 3D printed models. These models were viewed using the VR/AR interface of Z-Space software (zCORE, version 5.0, San Jose CA) The zSpace® system consists of a central processing unit (CPU), a 23.6 in, 1080-p high definition liquid crystal 3D stereoscopic display screen, a 3 button stylus with integrated haptic technology, and a set of polarized eye glasses with reflective sensors for tracking cameras (Fig. 4a-b). The stylus could manipulate a virtual slicing tool which projected through the VR model to obtain multiple planes. As the zSpace system does not use a head-mounted immersive display but a projected image viewed through glasses, there is some overlap with classical AR platforms in which there is a superimposed digital image on the real world. We retained the categorization of VR given the primary focus of the virtual image over the real background.

To achieve randomization, participants signed up for time slots unrestricted and ungrouped by any participant characteristics. Each consecutive participant was then assigned to be taught using either the VR models or the 3D models in an alternating pattern. Following consent, trainees and practitioners underwent individual 15-minute teaching sessions with us where we demonstrated features of a developmentally typical heart as well as a congenitally diseased heart (tetralogy of Fallot) using both VR and 3DP. The participants were trained in the same way regardless of whether they were being shown a developmentally typical or pathological heart. The 3DP demonstration included identification of key structures along the pre-set slicing planes. The VR demonstration included a brief demonstration on how to manipulate the VR model as well as how to use the slicing tool to obtain multiple cuts through each model. Participants then briefly explored each modality individually before filling out a short survey (Additional file 1) in which they identified which model (3DP or VR) they felt was more effective in enhancing their understanding of cardiac anatomy and associated defects. The survey included a binary summative assessment and a series of Likert scale questions addressing usefulness of each model type and degree of comfort with each modality. We deliberately chose to keep the questions concise and straightforward as the scope of this study was limited to initial impressions of understanding based on each modality.

Responses were compared using 2 way paired t-tests/ ANOVA or non-parametric tests based on distribution. Univariate regressions were performed to determine associations. In addition, Pearson correlation coefficients were calculated using covariance and standard deviation data to determine strength of relationships. All statistical analyses were performed using SPSS 19.0 (SPSS Inc., Chicago, IL). Statistical significance was defined as *p* < 0.05.

## Results

Twenty-seven pediatric residents and 3 nurse practitioners explored models of a developmentally typical heart and tetralogy of Fallot pathology (*n* = 30). The pediatric residents consisted of 7 interns, 12 second year residents and 8 third year residents. Overall, participants endorsed a greater degree of understanding with VR models (8.5 ± 1) compared with 3DP (6.3 ± 1.8) or traditional models of instruction (5.5 ± 1.5) *p* < 0.001 (Table 1).

**Table 1:** Subjective level of understanding by modality

Subjective level of understanding	Likert scale, 1–10 [mean, standard deviation]
VR models	8.5 ± 1
3DP	6.3 ± 1.8
Transitional models of instruction	5.5 ± 1.5
*p*-value	< 0.01

**Fig. 5:**
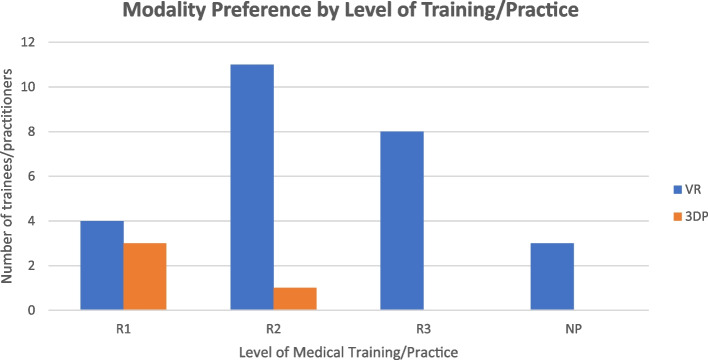
Preference of modality by level of training/practice

“Traditional models of instruction” refers to how participants are typically instructed on such subject matter in prior teaching sessions using 2D schematics as a subjective comparison point to the 3D modalities comprising this study. Most participants identified minimal prior exposure to VR (1.1 ± 0.4) or 3-D printed models (2.1 ± 1.5). Based on broad minimal exposure and sample size, no adjustment was made for previous exposure to VR or 3DP. Most participants expressed comfort with the use of modern technology at baseline (7.6 ± 2.1). “Modern technology” was not explicitly defined but implicitly alluded to the digital tools that form part of modern daily lived experience. By level of training, 4/7 (57%) interns preferred VR while 11/12 (92%) of second year residents and 8/8 (100%) of third year residents held a similar preference for VR (Fig. 5).

In the summative assessment, 87% (*n* = 26) expressed a preference for Virtual Reality models compared to 13% (*n* = 4) for 3DP. In addition, participants offered narrative elaboration on their preferences (Table 2).

**Table 2:** Resident/nurse practitioner comments on 3D vs VR learning experience

*“Virtual reality made it easier to visualize the heart from all planes. 3D Printed model was more restrictive.”*	
*“Seeing the 3D printed model helped me differentiate normal* vs *abnormal structures better.”*	
*“I think prospects for VR in pediatric medical education are great.”*	
*“I would like the Virtual Reality better if there were more colors than grey scale to follow flow patterns”*	
*“Virtual Reality models are beautiful and more intuitive than 2D models.”*	

## Discussion

Medical education within Pediatric Cardiology is increasingly recognizing that extrapolation of 3 dimensional structures from 2 D models, while possible and trainable, may not be the optimal way to teach trainees about the heart. Given the close relationship between spatial orientation and physiology that characterizes Pediatric Cardiology, effective instruction must provide dynamic visual representation. 3DP and VR have arisen as potential tools in this effort and the preponderance of evidence to date suggests benefits in regard to learner engagement [1, 7, 16–18]. Less robust evidence exists to compare the relative value of these representations. This question is important to address as the two approaches vary significantly in regard to questions of cost, preparation time, and portability which are key factors in the wider adoption of these approaches in curricula. It was this gap in educational efficacy between 3DP and VR that our study was designed to begin to address. By systematically comparing trainee experience with each modality side by side, a meaningful assessment was obtained to help guide further training efforts and studies.

The results were somewhat surprising in how definitively they skewed toward VR versus 3DP (87% vs 13% summative preference). We hypothesized that there would be a significant number of participants for whom the tactile and haptic qualities of 3DP models would make them preferable to VR models. For the few who in fact had this preference, these factors were mentioned. Also noted, however, was the limitation of the pre-determined cutting planes. In contrast, VR, had a slicing tool which offered multiple planes in virtually any orientation and was repeatedly cited as an appealing factor in narrative comments (Table 2). This ability to direct the learning experience more precisely is what a previous study on virtual skills learning identified as “presence” and “agency” [19]. However, the grey scale of the Virtual Reality model was mentioned as a limitation compared to the two colored 3DP models, suggesting there is a clarifying role for color differentiation regardless of modality. Other software interfaces have color options which likely will improve satisfaction even more.

An additional consideration in comparing these modalities is the effect of prior exposure, whether direct as in VR/3DP in CHD education or indirect as in other settings such as video game usage. Related to this exposure question is Roger and Cohen’s (2020) discussion of generational learning where he advocates for a framework that is best suited to the current generation of learners to include technology [20]. In our study, participants generally had limited prior exposure to either VR (1.1 ± 0.4) or 3DP (2.1 ± 1.5) but felt generally comfortable with modern technology (7.6 ± 2.1). We hypothesize that the minimal prior VR/3DP exposure limits the effect of this exposure on the preference of VR over 3DP; similarly comfort with daily modern technology may translate into increased ease with VR technologies or, alternatively, may be less applicable to the novel spatial challenges of these modalities. Novel features like pen wand navigation may have contributed to the appeal of VR.

Digging deeper into what it means to “like” or “prefer” a modality raises additional questions: did intrinsic parts of the educational process such as learning to use the VR wand offer an internal reward that made preference more likely? Less ambiguous is that experienced raters were essentially unanimous in their VR preference. This preference could reflect less of a need or desire on the part of early learners for dimensional data than expert learners.

The educational potential of Virtual Reality is certainly being explored in a number of other fields as well with potentially transferable principles. In nursing, for example, an intervention group who were taught a procedure using VR were able to perform more of these procedures in an hour compared to the control group [21]. However, these gains were not sustained 2 weeks after the initial study suggesting that some of the benefits that VR imparts may require tech “boosters” to be sustained. VR is also being employed in pharmacy education where dynamic applications are being explored such as tracking a drug as it proceeds through the body observing visually how it is changed at each stage [22]. Such dynamic 4D tracking can be applied to real time analysis of cardiac structures as described in a recent technological innovation review [23]. In the orthopedic domain, a study examining the impact of VR and 3D models on preoperative planning for humeral fracture repair found the use of these modalities led to shorter operative time and less blood loss than conventional methods [24].

Of critical importance in the ongoing evaluation of these modalities is to consider both objective effectiveness and feasibility. In a recent study examining the impact of VR on participant understanding of atrioventricular canals, no difference was found in post-test scores between the control group (desk-top computer) and intervention group (VR). However, the VR group did report a better learning experience and engagement level. Almost counterintuitively, the VR group also had a stronger correlation between their perceived strength of knowledge and their actual performance suggesting that this modality may have role in bridging the gap between perceived knowledge and actual knowledge [25]. In a counterexample, a study looking at the relationship between participant confidence of correctness and actual correctness in the virtual environment of a pre-surgical planning session found the correlation was low [26]. This finding may be related to the challenges of measuring depth and features in VR. There continues to be a need for rigorous studies that evaluate objective improvement in knowledge acquisition and spatial conceptualization which can be difficult to capture. Su et al., (2018) developed a controlled study examining the impact of 3D models in a medical student curriculum is a promising example [18]. By asking both subjective questions as well as fact based and spatial conceptualization questions in the post-test, this study was able to demonstrate improvement in knowledge acquisition more rigorously. In regard to 3DP, a recent review highlights the need to systematically examine if there are certain groups who may benefit more from such modalities [7].

Having demonstrated effectiveness, the final hurdle for the wider use of such modalities is feasibility. 3DP models are expensive and time consuming to prepare [17]. VR, depending on the interface, can also involve significant cost but lower technology iterations exist. If such factors as cost can be addressed, VR holds further promise given shareability. Such technological nimbleness and ability to share remotely is critical in an age where we witnessed a physical interaction standstill with the novel coronavirus (COVID-19). Further nuanced work can reveal where modalities like Augmented Reality (AR), which retains the capacity to still see the physical world, may be more optimal [27]. VR promises to not only make CHD education more effective, but may also have important global pediatric cardiology applications including the capacity to remotely train others in low and middle income countries (LMIC) where such work could be an important part of capacity building [28]. Such work would also form a robust response to the charge issued by the Lancet Independent Global Commission for the Education of Health Professionals for the twenty-first Century calling for “transformative learning” through the harnessing of technological innovations [29] .

## Limitations

This was a single center study with a modest number of participants. Results were self-reported and such perceptions in learning impact are by nature subjective. A comprehensive, objective post-test would more rigorously support improvements in learning and would be an important consideration for subsequent comparative studies. As the participants were primarily residents, these results are not necessarily generalizable to higher level trainees such as fellows. In addition, our group was primarily composed of medical residents; surgical residents may have derived a differential benefit from each representation. Again, a comparative study, perhaps with more complex lesions would be a promising future line of inquiry in this direction. With the global phrasing of our questions in the questionnaire, our conclusions were limited regarded perceived understanding of specific features of the tetralogy of Fallot heart for example. Along with including more complex lesions in the future, it will be important to ask more specific anatomic questions to assess understanding of pathology more precisely. Finally, it was challenging to fully account for the effect of prior “VR like”experience. While most participants endorsed minimal prior exposure to VR, VR like experiences in non-educational settings such as video games and mobile applications may have influenced their preference for this representation. Future studies can more specifically ask about these experiences as well as track if VR preference tracks along any demographic lines.

## Conclusion

Our study shows that, overall, VR was preferred over 3DP by pediatric residents and nurse practitioners for understanding cardiac anatomy and pathophysiology. Future comparative studies with objective assessments as well as explorations into questions of feasibility such as cost and portability will help to illuminate the full pedagogical value of these modalities.

## Supplementary Information

**Additional file 1.**

## Data Availability

Data not currently deposited but can be made available to interested parties.
