# Supplementary material for: Comparative effectiveness of virtual reality (VR) vs 3D printed models of congenital heart disease in resident and nurse practitioner educational experience
Source: 3D Print Med. 2023 Feb 11;9:2. doi: 10.1186/s41205-022-00164-6 (PMC9918815; doi:10.1186/s41205-022-00164-6)
Supplement: Supplementary file 1 — Additional file 1. [file 41205_2022_164_MOESM1_ESM.docx]

**Appendix 1: Medical Personnel Questionnaire**

| Resident (1) or Nurse Practitioner (2) | 1 | 2 |  |  |  |  |  |  |  |  |
| --- | --- | --- | --- | --- | --- | --- | --- | --- | --- | --- |
| If Resident, Level of Training  (1,2,3) | 1 | 2 | 3 |  |  |  |  |  |  |  |
| How much prior training/experience do you have with Virtual Reality cardiac models? | 1 | 2 | 3 | 4 | 5 | 6 | 7 | 8 | 9 | 10 |
| How much prior training/experience do you have with 3D printed cardiac models? | 1 | 2 | 3 | 4 | 5 | 6 | 7 | 8 | 9 | 10 |
| How was your level of understanding with traditional explanation? | 1 | 2 | 3 | 4 | 5 | 6 | 7 | 8 | 9 | 10 |
| How was your level of understanding with 3D printed model? | 1 | 2 | 3 | 4 | 5 | 6 | 7 | 8 | 9 | 10 |
| How was your level of understanding with Virtual Reality model? | 1 | 2 | 3 | 4 | 5 | 6 | 7 | 8 | 9 | 10 |
| Usefulness of 3D printed model | 1 | 2 | 3 | 4 | 5 | 6 | 7 | 8 | 9 | 10 |
| Usefulness of Virtual Reality model | 1 | 2 | 3 | 4 | 5 | 6 | 7 | 8 | 9 | 10 |
| Level of comfort with modern technology | 1 | 2 | 3 | 4 | 5 | 6 | 7 | 8 | 9 | 10 |
| Would you prefer a Virtual reality (1) or 3D printed model (2)? | 1 | 2 |  |  |  |  |  |  |  |  |
